# *C*-*Raf* deficiency leads to hearing loss and increased noise susceptibility

**DOI:** 10.1007/s00018-015-1919-x

**Published:** 2015-05-15

**Authors:** Rocío de Iriarte Rodríguez, Marta Magariños, Verena Pfeiffer, Ulf R. Rapp, Isabel Varela-Nieto

**Affiliations:** 1 Instituto de Investigaciones Biomédicas “Alberto Sols”, CSIC-UAM, Arturo Duperier 4, 28029 Madrid, Spain; 2 Centre for Biomedical Network Research (CIBERER), Institute of Health Carlos III (ISCIII), Madrid, Spain; 3 Departamento de Biología, Universidad Autónoma de Madrid, Darwin 2, 28049 Madrid, Spain; 4 Institute for Medical Radiation and Cell Research (MSZ), University of Würzburg, Versbacher Strasse 5, 97078 Würzburg, Germany; 5 Hospital La Paz Institute for Health Research (IdiPAZ), Madrid, Spain; 6 Present Address: Institute for Anatomy and Cell Biology, University of Würzburg, Koellikerstraße 6, 97070 Würzburg, Germany; 7 Present Address: Molecular Mechanisms of Lung Cancer, Max Planck Institute for Heart and Lung Research, Parkstr. 1, 61231 Bad Nauheim, Germany

**Keywords:** Programmed cell death, ERK, FoxG1, Inflammation, NIHL, Otic

## Abstract

The family of RAF kinases transduces extracellular information to the nucleus, and their activation is crucial for cellular regulation on many levels, ranging from embryonic development to carcinogenesis. B-RAF and C-RAF modulate neurogenesis and neuritogenesis during chicken inner ear development. C-RAF deficiency in humans is associated with deafness in the rare genetic insulin-like growth factor 1 (IGF-1), Noonan and Leopard syndromes. In this study, we show that RAF kinases are expressed in the developing inner ear and in adult mouse cochlea. A homozygous *C*-*Raf* deletion in mice caused profound deafness with no evident cellular aberrations except for a remarkable reduction of the K^+^ channel Kir4.1 expression, a trait that suffices as a cause of deafness. To explore the role of *C*-*Raf* in cellular protection and repair, heterozygous *C*-*Raf* ^+*/*−^ mice were exposed to noise. A reduced C-RAF level negatively affected hearing preservation in response to noise through mechanisms involving the activation of JNK and an exacerbated apoptotic response. Taken together, these results strongly support a role for C-RAF in hearing protection.

**Electronic supplementary material:**

The online version of this article (doi:10.1007/s00018-015-1919-x) contains supplementary material, which is available to authorized users.

## Introduction

The RAF-MEK-ERK pathway conveys growth factor signals from the cell surface to the nucleus to drive a wide variety of physiological outcomes [1]. RAF activities are central to embryonic development [2], cancer [1], stem-cell generation [3], and cell protection and regeneration [4, 5]. RAF serine/protein kinases transfer information to the MEK-ERK module through direct phosphorylation to ultimately facilitate transcriptional activity. RAF proteins also facilitate cell survival by directly interacting with other proteins such as 14–3–3, through mechanisms independent of their kinase activity [6–8].

In mammals, the RAF family of kinases consists of three members: A-RAF, B-RAF, and C-RAF. All have autoinhibitory, regulatory, and catalytic domains with phosphorylation activity [9–11]. RAF kinases have specific tissue and cell-type expression patterns. A-RAF is ubiquitous and its functions have been related to endocytic trafficking [12]. Accordingly, *A*-*Raf* knockout mice die around P20 due to neural defects and bowel distension [13]. Evolutionarily, B-RAF is the ancestral RAF kinase, and it is highly expressed in the nervous system [14]. Although the three RAF proteins can phosphorylate ERK, B-RAF is the most efficient one, and therefore, it plays a central role in the physiopathology of cell proliferation [15]. The first *Raf* gene was isolated from retroviruses [16] and was found to be closely associated with cancer [17]. C-RAF is also ubiquitous [18] and plays a role in cell survival [19].

The three RAF kinases have redundant but also specific functions, depending on the temporal and cellular contexts [20–22]. B-RAF and C-RAF play distinct roles in cell proliferation and survival, respectively. This distinction is the case during the early development of fish and chicken inner ears [23–26]. In mice, ERK1/2, the phosphorylation target of RAF kinases, is activated as part of the repair response to cochlear injury [27, 28]. Finally, deficiencies in IGF-1 and C-RAF have been implicated in some rare and severe human syndromes with defects that include deafness, such as *IGF1* deficit (OMIM **#**147440), Noonan (NS5; OMIM **#**611553), and Leopard (LPRD2; OMIM **#**611554) syndromes [7, 29–32]. To date, the mechanisms underlying the functions of C-RAF in mammalian auditory organs are poorly understood, and the impact of chronic C-RAF deficiency on hearing has not been investigated.

Sensory hair cells located in the organ of Corti are responsible for sound reception and information transmission to the brain. Hair cells are bathed in a potassium-rich fluid called endolymph, whose composition is essential for hair-cell depolarization and stimuli transduction. Mutations in potassium channels are well-defined genetic causes of deafness [33, 34]. Stria vascularis cell types, fibrocytes of the spiral ligament, and other cochlear cells contribute to potassium recycling and endolymph homeostasis [35]. Environmental exposure to noise causes hearing loss, but the extent of damage has an unknown genetic component [36]. Noise-induced hearing loss (NIHL) involves alterations in the ion balance [37, 38] and triggers a JNK-mediated inflammatory and pro-apoptotic response. Although the precise mechanisms underlying the predisposition to noise damage are not well defined, defects at the critical nodes of cell survival and repair responses are likely to contribute.

In this study, we investigated the role of C-RAF in the physiopathology of hearing loss using wild-type *C*-*Raf* ^+*/*+^, heterozygous *C*-*Raf* ^+*/*−^, and *C*-*Raf* ^−*/*−^ null mice. The three RAF kinases are expressed and active during inner ear development. The complete elimination of C-RAF caused profound deafness, but this was not sufficient to cause cellular malformations in the cochlea. As most C-*Raf* null mutants die at embryonic or early postnatal ages, heterozygous mice were studied as a model of chronic C-RAF deficiency. Heterozygous mice exhibited normal hearing but experienced exacerbated injury in response to noise exposure that was associated with basal JNK activation and an increased rate of apoptosis. Our data strongly support the hypothesis that C-RAF activity is essential for protection and repair of the auditory organ.

## Materials and methods

### Mouse handling and genotyping

*C*-*Raf* ^−*/*−^ mice were generated [39] and backcrossed to an Ola:MF1 genetic background that allows postnatal survival [40, 41]. Null (*C*-*Raf* ^−*/*−^), heterozygous (*C*-*Raf* ^+*/*−^), and wild-type (*C*-*Raf* ^+*/*+^) littermates used were maintained on a Ola:MF1 genetic background. Null mice died between postnatal (P) days 20–40 (postnatal survival rate = 1 %) due to massive liver apoptosis [19, 42]. No differences between male and female mice were observed, and both were used in this study at the embryonic (E) and postnatal (P) ages indicated in the text. Mice genotypes were determined using the REDExtract-N-Amp Tissue PCR Kit (Sigma-Aldrich) with primer pairs for detection of the wild-type *C*-*Raf* allele [5′-ACAGAAAGTGTAGCTGCAGTGA-3 and 5′-ATTGATTTGATTGCCAGGTATGAT-3′ (335-bp band)] and the neomycin cassette [5′-ACAGAAAGTGTAGCTGCAGTGA-3 and 5′-TGCGTCCAATCCATCTTGTTCAA)-3′ (450-bp band)]. Animal care procedures and use were approved by the Bioethics Committee of the CSIC. Experimental procedures were conducted in accordance with European Union (2010/63/EU) and Spanish R&D (53/2013) legislations.

### In vivo evaluation of auditory brainstem response (ABR), noise exposure, and vestibular function

Three genotypes of P20–60 mice were anesthetized by i.p. administration of ketamine (Imalgene© 1000, Merial; 100 mg/kg) and xylazine (Rompun© 2 %, Bayer Labs; 4 mg/kg). Hearing was evaluated by registering the auditory brainstem response (ABR) as described [43]. Click and 8–40 kHz tone-burst stimuli (0.1 and 5 ms duration, respectively) were generated with SigGenRP™ software (Tucker-Davis Technologies, Alachua, FL, USA). Stimuli were presented monaurally at 30 or 50 pulses per second each, from 90 to 10 dB relative to sound pressure level (dB SPL) in 5–10 dB SPL steps, and the electrical response was amplified, recorded, and averaged (1000 and 750 stimulus-evoked responses for click and tone burst, respectively), using BioSigRP™ software. Hearing thresholds (dB SPL) and wave latencies (ms) were calculated based on the ABR waves that were registered as reported [44]. When indicated, P60 heterozygous and wild-type mice were exposed to high frequency-enriched noise for 30 min at an intensity of 110 dB SPL in a sound-proof reverberant chamber [45]. The vestibular function of P20–40 null, heterozygous and wild-type littermates was tested as reported, in a series of simple tests adapted from standard protocols ([46]; http://empress.har.mrc.ac.uk/), as previously described [47]. Briefly, mice were observed for behavioral aberrations associated with vestibular disorders such as circling, head bobbing, or abnormal gait. Then the following behaviors were sequentially evaluated: (i) the ability of mice to reach a horizontal surface and the presence of abdominal forward curling; (ii) their performance in contact righting and air righting tests to evaluate the mice’s ability to reorient their bodies from an inverted position; and (iii) their performance in a swimming test to discover abnormal swimming behaviors such as vertical, circular or side swimming, or immobile floating.

### Middle ear dissections

Three P20 mice of each genotype were administered a lethal dose of pentobarbital (Dolethal, Vétoquinol). The ossicles—the malleus, incus, and stapes—of the middle ear were dissected. Microphotographs of these structures were taken using a digital camera connected to a Leica MZ8 stereo microscope (Leitz).

### Histology and immunohistochemistry

Cochleae were dissected, photographed, and fixed overnight in 4 % paraformaldehyde in PBS (4 °C), decalcified in 0.3 M EDTA (pH 6.5) for 8 days, and embedded in paraffin, celloidin, or gelatin. Sections of the paraffin-embedded (5 μm) or celloidin-embedded (2 μm) tissues were stained using hematoxylin/eosin and cresyl violet, respectively, to study cochlear cytoarchitecture or were subjected to immunohistochemistry, as described [48, 49]. After incubation with the primary antibody overnight (4 °C) (Supplementary Table S1), sections were incubated with biotinylated secondary antibodies (Chemicon; dilution 1:200) for 2 h and processed using an ExtrAvidin-peroxidase conjugate solution (1:200, Sigma). Antibody binding was visualized using DAB, and sections were mounted in Entellan^®^ (Merck Chemicals) for observation using a Zeiss Axiophot microscope equipped with an Olympus DP70 digital camera. Cryosections (10 μm) were incubated as above and then with Alexa Fluor-conjugated secondary antibodies for 2 h (RT). Sections were mounted in Prolong Gold containing DAPI (Invitrogen) and visualized using a fluorescence microscope (Nikon 90i, Tokyo, Japan). The intensity of neurofilament, synaptophysin, Kir4.1, myelinP0, KCNQ1, immunofluorescence, Sox2, and myosin VIIa positive cells were determined and counted, respectively, in 4–12 equivalent sections prepared from at least 3 mice of each genotype and experimental group. Quantifications were performed in a region of interest (ROI) in each cochlea from base to apex using ImageJ software (National Institutes of Health, Bethesda, MD, USA) [50].

## TUNEL

Apoptosis was evaluated by TdT-mediated dUTP nick-end labeling (TUNEL) using the ApopTag kit (Millipore/S7101). Cryosections (10 μm) were fixed using 1 % PFA pH 7.4 for 10 min (RT). The sections were post-fixed in ethanol/acetic acid (2:1, by vol.) for 5 min (−20 °C), incubated with the TdT enzyme (37 °C) for 1 h and processed using an anti-digoxigenin conjugate. Apoptotic cells were visualized using a peroxidase substrate solution. The TUNEL-positive nuclei in the organ of Corti in 6 (non-exposed wild type), 10 (wild type exposed to noise), 8 (non-exposed heterozygous), and 10 (heterozygous exposed to noise) sections prepared from three mice of each genotype and the experimental group were quantified using NIH ImageJ software as described above.

### Protein extraction and Western blotting

Proteins from frozen cochleae of at least three mice/experimental group were extracted using a Ready Protein Extraction Kit (BioRad). Cochleae were lysed in 100–250 μl of extraction buffer containing 0.01 % protease- and phosphatase-inhibitor cocktails and 0.01 % TBP (Sigma-Aldrich). Protein concentration was determined with the RC DC Protein Assay kit (BioRad) using bovine serum albumin (BSA) as the standard. Equal amounts of cochlear proteins were subjected to gel electrophoresis (Any kD Mini-PROTEAN-TGX, BioRad) and transferred to PVDF membranes (0.2-µm, BioRad) using a Bio-Rad Trans Blot TURBO apparatus. After incubation with a blocking solution, the membranes were probed overnight (4 °C) with the primary antibodies indicated in Supplementary Table S1. Membranes were then incubated with a peroxidase-conjugated secondary antibody for 1 h (RT), and the bands were visualized using Clarity™ Western ECL Substrate (BioRad/170–5060). Images of the blots were captured using an ImageQuant LAS4000 mini digital camera (GE Healthcare Bio-Sciences), and densities of the immunoreactive bands were quantified by densitometry using ImageQuant TL software (GE Healthcare Bio-Sciences). Different exposure times were used to ensure that the bands were not saturated.

### Quantitative RT-PCR

RNA was isolated using RNeasy (Qiagen) from 1–2 cochleae; its integrity and concentration were assessed using an Agilent Bioanalyzer 2100 (Agilent Technologies). At least, three mice per condition were used. cDNA was then generated by reverse transcription (High Capacity cDNA Reverse Transcription Kit; Applied Biosystems) and gene expression analyzed in triplicate by qPCR using TaqMan^®^ Gene Expression Assay kits (Applied Biosystems). The following probes were used: *Foxm1* (Mm00514924_m1), *Foxg1* (Mm02059886_s1), *Gap43* (Mm00500404_m1), *Gmf*-*b* (Mm01322969_m1), *Igf1r* (Mm00802831_m1), *Mapk14* (Mm00442498_m1), *Mash1* (Mm03058063_m1), *Mef2d* (Mm00504931_m1), *Ntn1* (Mm00500896_m1), *p27kip1/Cdkn1b* (Mm00438168_m1), *A*-*Raf* (Mm00550186_m1), *B*-*Raf* (Mm01165837_m1), *C*-*Raf* (Mm00466513_m1), and *Sox2* (Mm03053810_s1). PCR was performed on an Applied Biosystems 7900HT Real-Time PCR System using eukaryotic 18S and RPLP0 rRNA as the endogenous housekeeping genes. Relative quantification values were calculated using the 2^−ΔΔCt^ method, and data were expressed as the mean log_10_RQ values [51].

### Statistical analysis

Data analysis was performed by running the Student’s *t* test using SPSS v19.0 software (SPSS Inc., Chicago, IL, USA). Post hoc multiple comparison analyses included the Bonferroni test when equal variances were assumed to exist. Data were expressed as mean values ± SEM. Results were considered significant at *P* < 0.05.

## Results

### RAF kinases are expressed in the developing and postnatal mouse inner ear

**Fig. 1:**
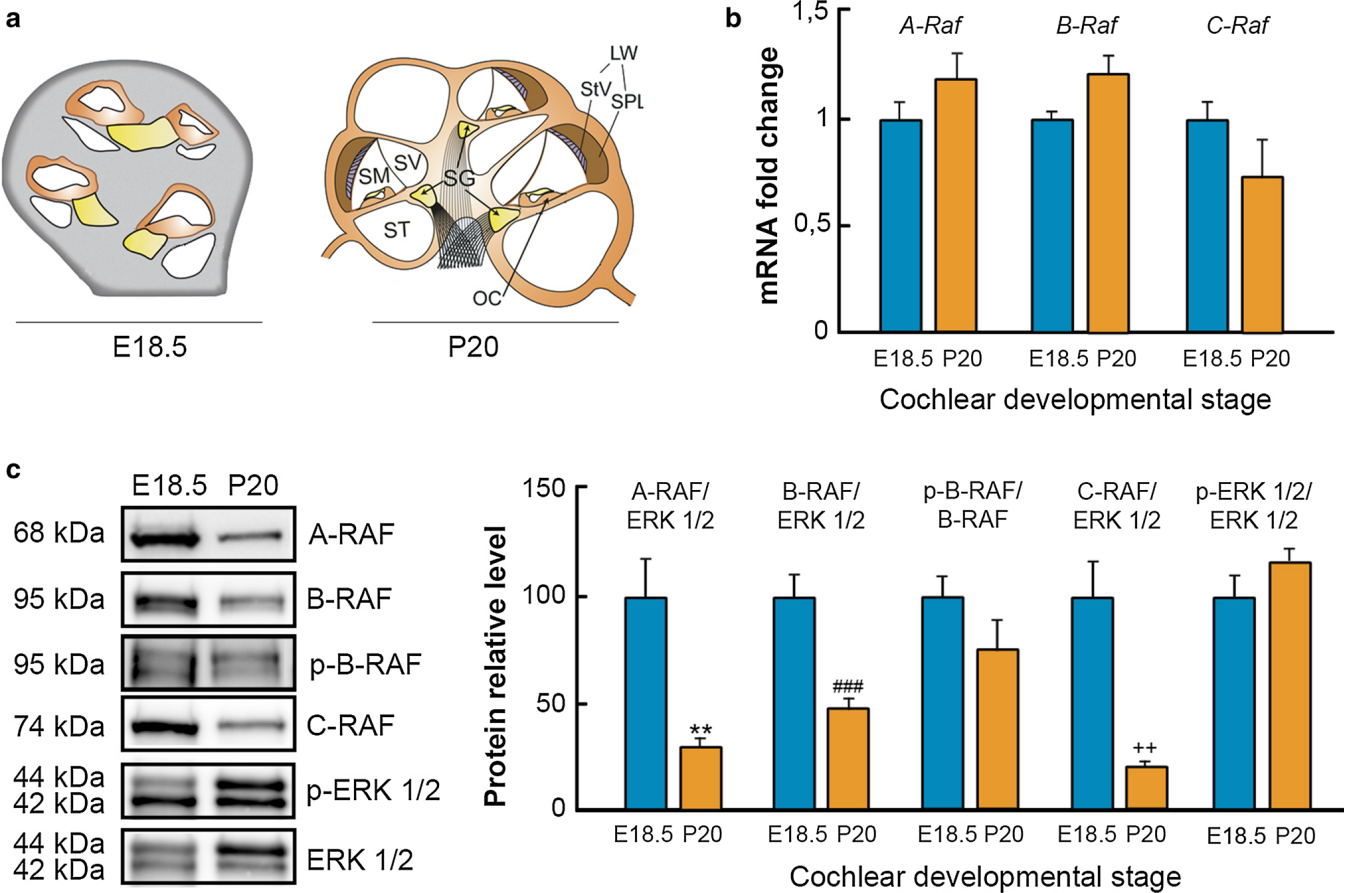
RAF kinases are expressed in the cochlea. **a** Schematic illustration comparing cochlear cross sections of E18.5 and P20 mice. *SV* Scala vestibuli, *SM* Scala media, *ST* Scala tympani, *OC* organ of Corti, *SG* Spiral Ganglion, *LW* Lateral wall, *StV* Stria vascularis, *SPL* Spiral ligament. **b** *A*-*Raf*, *B*-*Raf* and *C*-*Raf* mRNA expression levels in E18.5 and P20 cochleae were analyzed using RT-qPCR. 18s RNA was used as the endogenous control gene. At least, three mice from each stage were evaluated in triplicate. Data were normalized to the E18.5 levels and expressed as mean ± SEM of 2^−ΔΔCt^. The significance of the differences was evaluated using the Student’s *t* test. **c** A-RAF, B-RAF, C-RAF, and p-ERK1/2 levels in cochlear lysates were analyzed by Western blotting and normalized using ERK levels. p-B-RAF was normalized using B-RAF level. A representative sample blot from at least three mice in each stage is shown, and the average values obtained from densitometric measurements are plotted in *bar graphs*. The results are expressed as mean ± SEM. The significance of the differences was evaluated using Student’s *t* test. **P < 0.01 versus A-RAF E18.5; ^###^ *P* < 0.001 versus B-RAF E18.5; and ^++^ *P* < 0.01 versus C-RAF E18.5

To analyze temporal expression of RAF kinases, quantitative RT-PCR was performed using E18.5 and P20 cochleae (Fig. 1a, b). *A*-*Raf*, *B*-*Raf*, and *C*-*Raf* transcripts were similarly expressed at both ages, whereas RAF protein levels were significantly reduced at P20 (Fig. 1c). Interestingly, the p-B-RAF/B-RAF ratio was not reduced in the P20 cochlea when compared to that observed at E18.5. Accordingly, the phosphorylation level of its main target, ERK1/2, was not significantly changed (Fig. 1c).

### *C*-*Raf* null mice presented profound sensorineural deafness

**Fig. 2:**
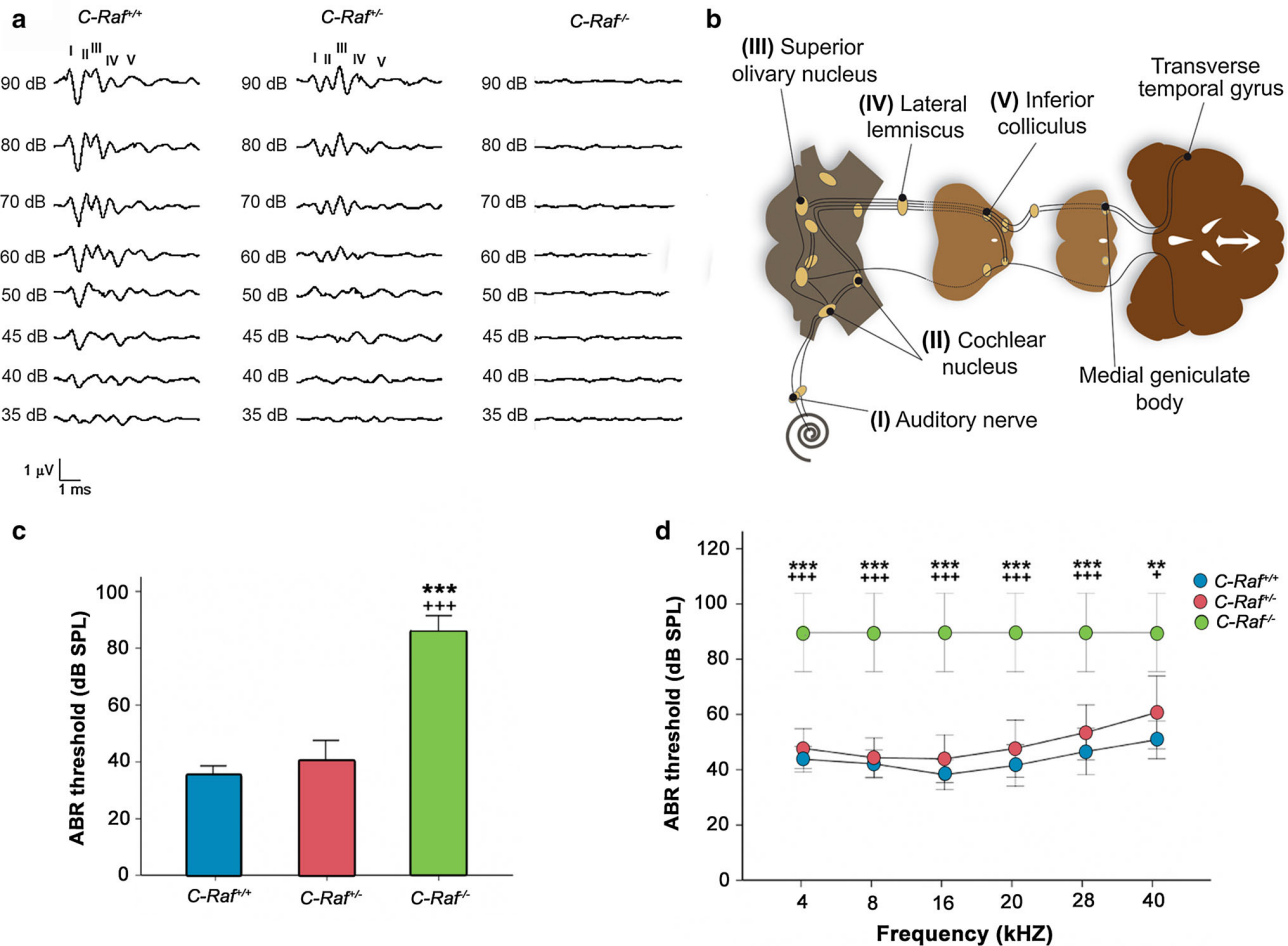
*C*-*Raf* null mice have profound bilateral sensorineural deafness. **a** Plots show representative ABR recordings of wild-type, heterozygous, and null mice. **b** The typical ABR profile consists of five waves corresponding to the different hearing processing centers: *I* auditory nerve; *II* cochlear nucleus; *III* superior olivary nucleus; *IV* lateral lemniscus; *V* inferior colliculus. **c** Average ABR thresholds for click stimuli in wild-type (*n* = 9), heterozygous (*n* = 13) and null mice (*n* = 4). **d** ABR thresholds in response to tone-burst stimuli in wild-type (*n* = 9), heterozygous (*n* = 13) and null mice (*n* = 4). Null mice showed severe sensorineural hearing loss. The significance of the differences was evaluated using Student’s *t* test. ***P < 0.001 versus wild type and ^+++^ *P* < 0.001 versus heterozygous

The ABR of the three genotypes was determined in 3- to 5-week-old mice (Fig. 2a, b), and demonstrated that wild-type and heterozygous mice had similar thresholds (35 ± 4 and 40 ± 5 dB SPL, respectively; Fig. 2c). In contrast, null mice showed no auditory response, even at the highest sound intensity tested (90 dB SPL; Fig. 2c). Frequency audiograms (Fig. 2d) and wave latencies (data not shown) were also studied in the three genotypes. Null mice were profoundly deaf but showed a normal response to standard vestibular screening tests (data not shown).

### Normal cytoarchitecture and aberrant Kir4.1 channel expression in *C*-*Raf* null cochlea

**Fig. 3:**
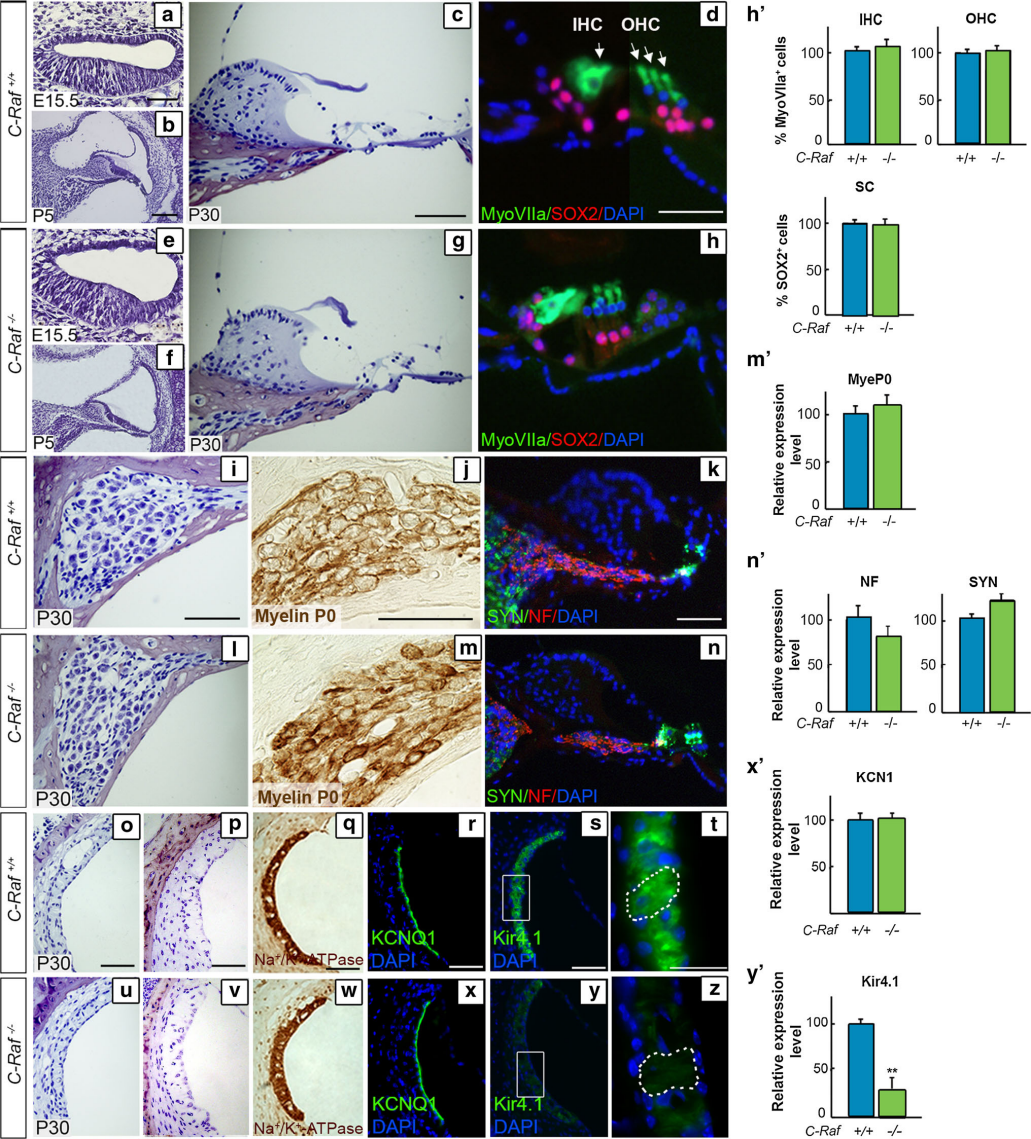
Characterization of *C*-*Raf* null mice cochlear cytoarchitecture and cell-type markers showing reduced levels of the Kir4.1 potassium channel, (**a**–**c**, **e**–**g**). Representative microphotographs of cross sections from wild-type and null mouse cochleae at E15.5, P5 and P30 (**d**, **h**, **h′**). The degree of Myosin VIIa (*green*) immunostaining/μm^2^ (outer and inner hair cells, OHC and IHC, respectively) and of SOX2 (*red*) (supporting cells, SC) was quantified in the organ of Corti (**i**, **l**). Neuronal distribution in the spiral ganglion was similar in P30 wild-type and null mice. Quantification of the intensity/μm^2^ of myelin P0 (**j**, **m**, **m′**), neurofilament (NF, *red*) and synaptophysin (SYN, *green*) (**k**, **n**, **n′**) staining showed no differences between genotypes. Representative microphotographs of paraffin-embedded (**o**, **u**) and semi-thin celloidin-embedded (**p**, **v**) cross sections of the stria vascularis from P30 wild-type and null mice (**q**, **r**, **w**, **x**, **x′**). Immunostaining of Na^+^/K^+^-ATPase and KCNQ1 channels in sections of wild-type mice and null mouse cochlea (**s**, **t**, **y**, **z**, **y′**). The Kir4.1 potassium channel expression level, also known as KCNJ10, was reduced in intermediate cells of null mice. Quantification of the immunofluorescence signal/μm^2^ was done using ImageJ software. Data were obtained from 4 to 12 sections of at least three mice from each genotype and plotted in bar graphs as the mean ± SEM relative to wild-type values. The significance of the differences was evaluated using Student’s *t* test: **P < 0.005 versus wild type. *Scale bars* 10 μm (**d**, **h**, **t**, **z**); 25 μm (**a**–**c**, **e**–**g**, **i**–**n**, **o**–**s**, **u**–**y**)

The general anatomy of the middle and inner ears of null mice was normal (data not shown), in striking contrast with the reported 70 % reduction in body size [39]. The general cochlear morphology of wild-type, heterozygous (data not shown), and null mice was entirely comparable at the selected embryonic and early postnatal stages (Fig. 3a, b, e, f). The outer and inner hair cells of the adult organs of Corti in the mutant mice had a normal appearance (Fig. 3, compare panels c, d with g, h), as confirmed by the pattern of myosin VIIa expression (h′). The number of supporting cells in the various genotypes was also similar, as indicated by transcription factor SOX2 expression (Fig. 3d, h, quantification shown in h′). Histological analysis of spiral ganglia showed no differences in cellular organization among the genotypes (Fig. 3i, l). We reported aberrant myelination for *Igf1* and *Irs2* mutant mice genes [30, 48], and therefore, the expression of myelin P0 was examined, but no significant differences were found among the genotypes (Fig. 3j, m, quantification shown in m′). Neurofilament and synaptophysin antibodies were used to examine the afferent fibers and the synaptic regions, respectively, but again there were no measurable differences among the genotypes (Fig. 3k, n, quantification shown in n′).

Histological examination of the stria vascularis in sections of 1-month-old wild-type, heterozygous (data not shown) and null cochleae revealed an apparently normal morphology and a similar capillary network in the three genotypes (Fig. 3, compare panels o, p with u, v). The stria vascularis is formed by marginal, intermediate-melanocytes, and basal cell layers. To further study this structure, the expression of cell-type specific channel proteins was assessed (Fig. 3, compare panels q–t with w–z). There were no differences in the Na^+^/K^+^-ATPase and KCNQ1 expression levels among the genotypes (Fig. 3, compare panels q–r with w–x, quantification shown in x′). In contrast, the potassium rectifying Kir4.1 channel expression level was significantly reduced, by 75 %, in null mice (Fig. 3s, y, quantification shown in y′). To exclude a migration defect, the intermediate cells were positively identified in null mice (Fig. 3t, z, dotted lines).

Functional tests revealed no obvious anatomical or cellular defects in the vestibular system of null mice (data not shown).

### Molecular profiling of the E18.5 *C*-*Raf* null cochlea showed up-regulation of *FoxG1*

**Fig. 4:**
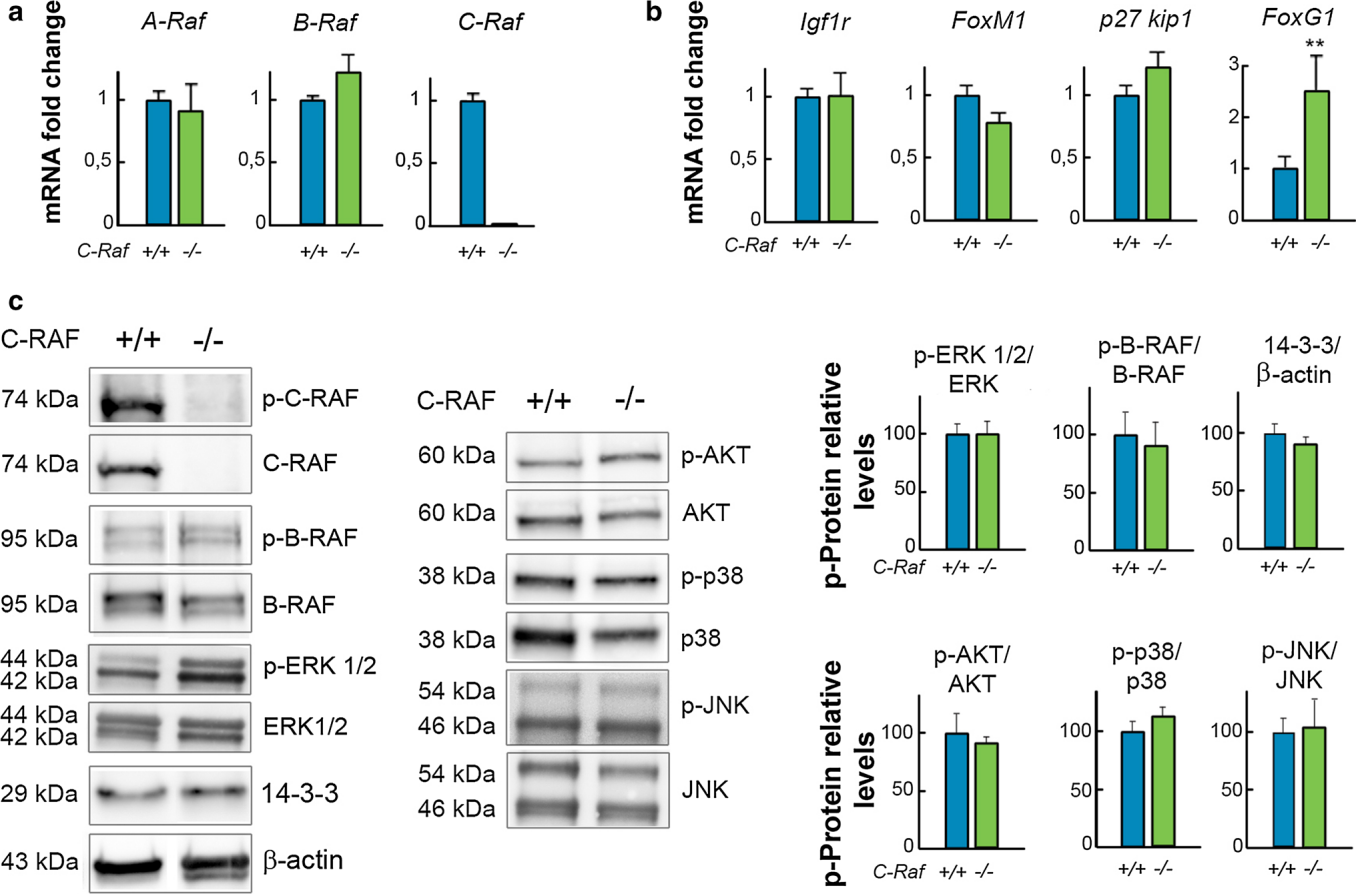
Expression of FoxG1 is up-regulated in *C*-*Raf* null mice cochleae. mRNA levels of (**a**) *A*-*Raf*, *B*-*Raf*, and *C*-*Raf* and (**b**) *Igf1r, FoxM1*, *p27 kip,* and *FoxG1* in E18.5 wild-type (*blue bars*) and null (*green bars*) cochleae were analyzed using RT-qPCR. 18s RNA was used as the endogenous control gene. At least, three mice from each genotype were evaluated in triplicate. Data were normalized to the levels of wild-type mice and were expressed as mean ± SEM of 2^−ΔΔCt^. The significance of the differences was evaluated using Student’s *t* test. **P < 0.005 versus wild type. **c** C-RAF, p-B-RAF, p-ERK 1/2, and 14–3–3 levels in wild-type and null cochleae were analyzed by Western blotting using cochlear lysates and were normalized to B-RAF, ERK 1/2, and β-actin levels, respectively. p-AKT, p-p38, and p-JNK levels were normalized with respect to total levels of their respective kinases. A representative blot of samples obtained from at least three mice of each genotype is shown. Densitometric average values are shown in the histogram. Results are expressed as mean ± SEM. The significance of the differences was evaluated using Student’s *t* test

Expression levels of thirteen cochlear genes in wild-type and null E18.5 mice were compared using RT-qPCR. Selected genes shown include those coding for RAF family members, *A*-*Raf, B*-*Raf, and C*-*Raf* (Fig. 4a), to test their potential up-regulation to compensate *C*-*Raf* absence. Other potential candidates for phenotype compensation are IGF-1 downstream signaling targets, including *Igf1r*, *FoxM1*, *p27kip1, Mapk14* [52], *Gap43*, and *Ntn1* [53] (Fig. 4 b; Supplementary Table S2). Finally, a panel of genes was tested to study if there was a neurodevelopmental alteration associated with the functional phenotype such as *FoxG1* [54], *Sox2* [55] (Fig. 4b; Supplementary Table S2), *Mef2D*, *Mash1* [52], and *Gmf*-*b* [56] (Supplementary Table S2). Expression levels of these genes were similar in both genotypes, with the exception of *FoxG1*, a neuronal pro-survival transcription factor [54, 57] that increased 2.5-fold in the null cochlea (Fig. 4b).

Next, we evaluated the levels and phosphorylation state of C-RAF, B-RAF, ERK1/2, and the 14–3–3 adapter proteins, as well as those of the PI3K/AKT, p38, and JNK kinases. There were no differences between the genotypes (Fig. 4c), which confirmed that the *C*-*Raf* deletion is not sufficient to halt inner ear development.

To further understand the role of C-RAF in adult hearing and given that *C*-*Raf* ^−*/*−^ null mice die in the first 2 months of life, *C*-*Raf* ^+*/*−^ heterozygous mice were used for further studies. However, two-month-old *C*-*Raf* ^+*/*−^ heterozygous mice showed normal baseline hearing and cochlear structure. The expected roles of C-RAF in cell self-repair and survival prompted us to study their response to stress.

### *C*-*Raf* heterozygous adult mice showed an increased susceptibility to NIHL

**Fig. 5:**
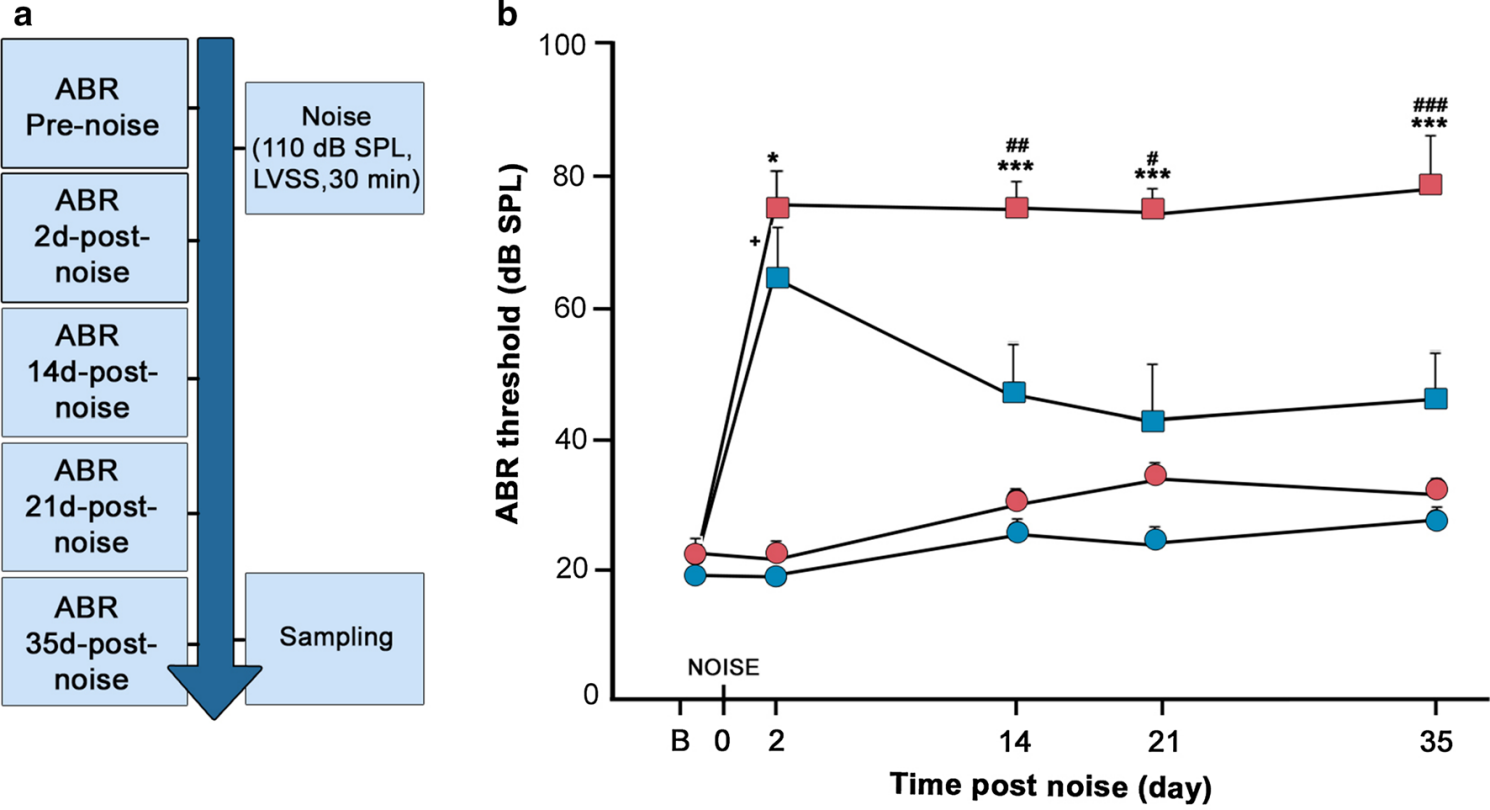
*C*-*Raf* heterozygous mice showed increased susceptibility to noise-induced hearing loss. **a** Scheme of the experimental design. Briefly, ABR thresholds were determined for the two experimental groups of 2-month-old wild-type (*n* = 16) and heterozygous (*n* = 15) mice. Next, one group of mice was exposed to moderate noise of 110 dB SPL (2–22 kHz) for 30 min in a sound-proof reverberant chamber. ABR evaluations were repeated on days 2, 14, 21, and 35. **b** Auditory click thresholds of both experimental groups were measured. Data from non-exposed mice (*n* = 4/genotype) are shown in *circles,* whereas data from noise-exposed (*n* = 4/genotype) mice are shown in *squares*. Wild-type (*n* = 4, *blue*) and heterozygous (*n* = 4, *red*) mice showed similar ABR-threshold increases 2 days after noise exposure. Wild-type mice (*blue squares*) gradually recovered their hearing, but heterozygous mice (*red squares*) did not. ABR thresholds of non-exposed mice from either genotype were not modified during the experiment. The significance of the differences was evaluated using Student’s *t* test: ^+^ *P* < 0.05 versus non-treated wild type; ^#^ *P* < 0.05 versus wild type exposed to noise; ^##^ *P* < 0.01 versus wild type exposed to noise; ^###^ *P* < 0.001 versus wild type exposed to noise; *P < 0.05 versus non-treated heterozygous; ***P < 0.001 versus non-treated heterozygous

P60 wild-type and *C*-*Raf* ^+*/*−^ heterozygous mice were noise challenged (Fig. 5a). Wild-type mice showed a temporary 40 dB ABR-threshold shift that was partially recovered 35 days later (Fig. 5b). Hearing recovery in wild-type mice was evident as early as 14 days after injury. In contrast, heterozygous mice showed a 60 dB ABR-threshold shift and no recovery 35 days later (Fig. 5b).

### Altered cytoarchitecture and cell-type expression markers in noise-exposed *C*-*Raf* heterozygous cochlea

**Fig. 6:**
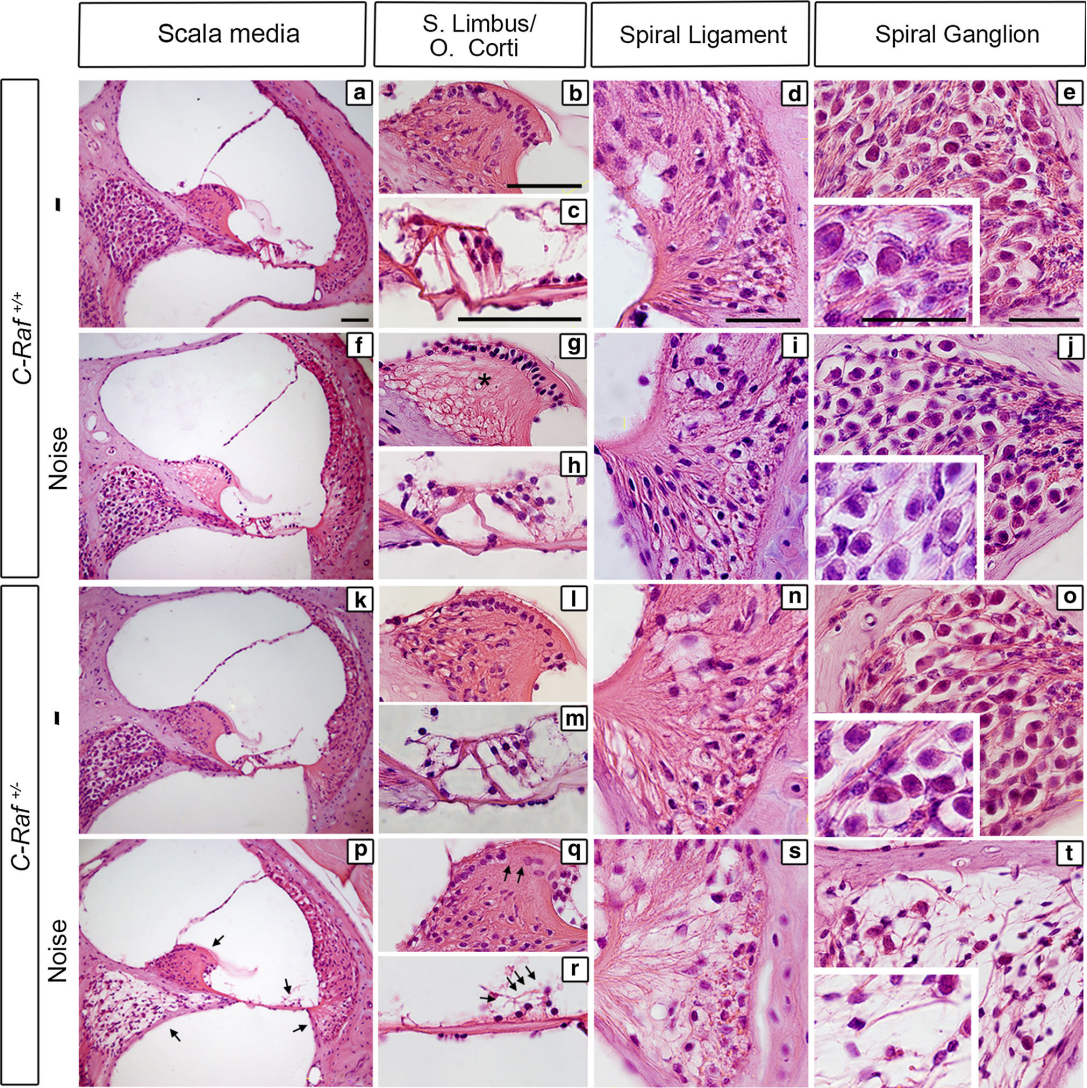
*C*-*Raf* heterozygous mice exhibited severe histological alterations in the organ of Corti and the spiral ganglion following noise exposure. **a**–**e** Histological cross sections of non-exposed wild-type mice. **f**–**j** Wild-type mice exposed to noise showed a reduction in the number of fibrocytes in the spiral limbus (**g**, *asterisk*), no other cellular alterations were observed, and the general cytoarchitecture was similar to that of non-treated wild-type mice. **k**–**o** Heterozygous mice not exposed to noise showed a cellular phenotype identical to wild-type mice. **p**–**t** Heterozygous mice exposed to noise showed the following alterations: loss of fibrocytes in the spiral limbus (**q**, *arrows*); loss of hair cells and supporting cells at the organ of Corti (**r**, *arrows*); loss of spiral ligament fibrocytes (**s**); and the spiral ganglion showed a drastic reduction in neuronal density (**t**). *Scale bars* 25 µm

Histological analysis of non-exposed wild-type (Fig. 6a–e) and heterozygous mice (Fig. 6k–o) showed normal cochlea during the study. 35-day post-noise exposure wild-type animals showed a reduction of the fibrocyte population in the central region of the spiral limbus but no other evident cellular alterations (Fig. 6f–j, asterisk in g). In contrast, heterozygous noise-exposed mice showed generalized cochlear damage (Fig. 6p–t). The interdental cells of the spiral limbus were absent (Fig. 6q, arrows), and the organ of Corti had a collapsed tunnel of Corti and a low density of hair cells and supporting cells (Fig. 6r, arrows). Moreover, the spiral ligament had a low density of fibrocytes with evidence of cellular debris within the fibrocyte type-IV region (Fig. 6s). Finally, the spiral ganglion was affected to different degrees, in both loss of both fibers and cellularity, as demonstrated by the increase in size of intercellular spaces (Fig. 6t).

**Fig. 7:**
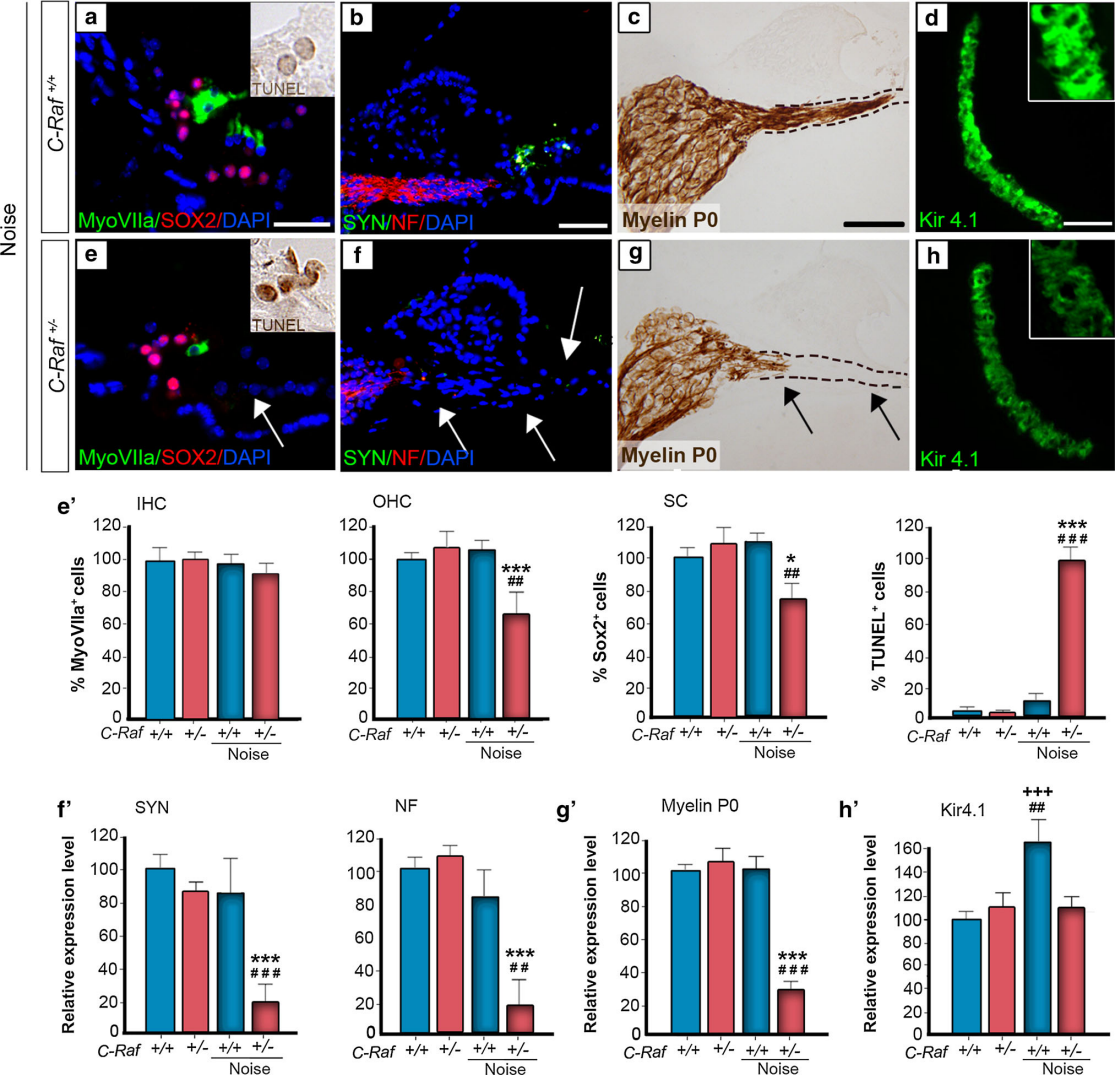
*C*-*Raf* heterozygous mice exhibited severe cellular alterations in the organs of Corti and spiral ganglion following noise exposure. **a**, **e**, **e′** Wild-type and heterozygous mice exposed to noise showed different immunostaining patterns. Myosin VIIa (*green*) positive outer hair cells and SOX2 (*red*) positive supporting cells showed 40 and 25 % reductions, respectively, in heterozygous mice. In contrast, TUNEL staining 2 days after noise exposure (*insets* **a**, **e**) showed a 95 % increment in the noise-exposed heterozygous mice with respect to any other experimental group (**e′**). **b**, **c**, **f**, **g**, **f′**, **g′** Neuronal markers NF (*red*), SYN (*green*) and myelin P0 (*brown*) evidenced neuronal and nerve fiber loss. Quantification of staining/μm^2^ showed that only heterozygous mice exposed to noise presented a general reduction. **d**, **h**, **h′** Immunostaining for the Kir4.1 potassium channel (*green*) in the stria intermediate cells increased by 60 % in the wild-type but not in the heterozygous mice after noise exposure. The intensity of the immunofluorescence per μm^2^ of the regions was quantified using ImageJ software. Data were obtained from 4 to 12 sections of at least three mice from each genotype and are shown relative to those of non-exposed wild-type mice as mean ± SEM. The significance of the differences was evaluated using Student’s *t* test: ^+++^ *P* < 0.001 versus non-treated wild type; *P < 0.05 versus non-treated heterozygous; ***P < 0.001 versus non-treated heterozygous; ^##^ *P* < 0.01 versus wild type exposed to noise; ^###^ *P* < 0.001 versus wild type exposed to noise; *Scale bars* 10 µm (**a**, **e**); 25 µm (**b**–**d**, **f**–**h**)

The severity of the cellular alterations observed in heterozygous mice exposed to noise was confirmed by evaluating the expression of cell-type markers 35 days after noise exposure. Myosin VIIa and SOX2 immunostaining indicated, respectively, a 25 % significant reduction in the population of supporting cells and a 40 % decrease in the number of outer hair cells in noise-exposed heterozygous mice compared to all the other experimental groups (Fig. 7, compare panel a with e, arrow, quantification shown in e′). Accordingly, a 95 % increase in the number of apoptotic TUNEL^+^ cells was evident 2 days after noise exposure in heterozygous cochlea (Fig. 7a, e, insets; TUNEL quantification in e′). Neurofilament and myelin P0 expression decreased by 80 and 70 %, respectively, confirming neuronal degeneration of heterozygous spiral ganglia. Hair cell synapses were also significantly affected, as indicated by the 80 % decrease of synaptophysin labeling (Fig. 7, compare panels b, c with f, g, quantification shown in f, g′). Finally, only wild-type mice exhibited a 60 % increase in the Kir4.1 expression level in response to noise (Fig. 7d–h, quantification in h′).

### *C*-*Raf* heterozygous mice showed basal activation of apoptotic signaling pathways

**Fig. 8:**
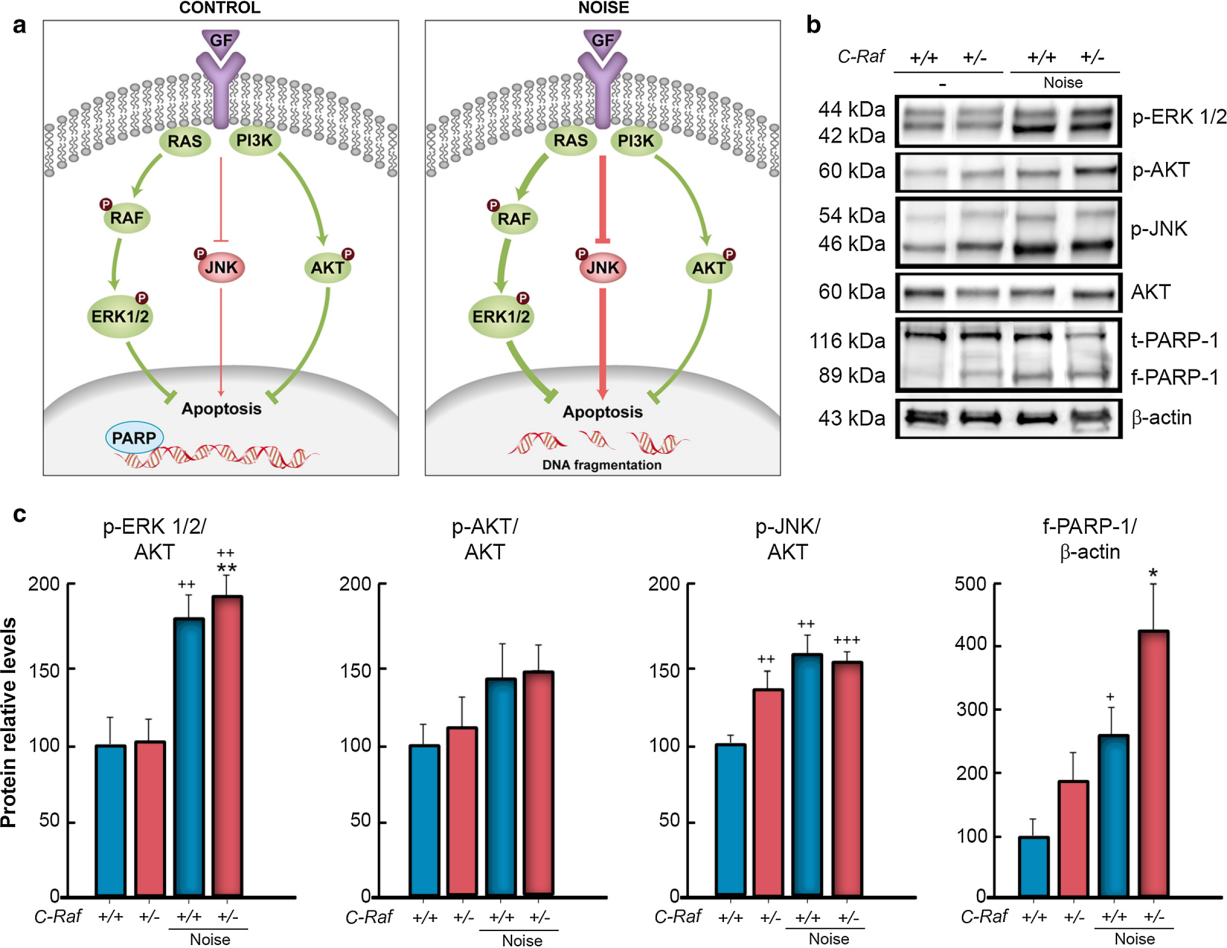
*C*-*Raf* heterozygous mice showed basal JNK activation and increased PARP fragmentation after noise exposure. **a** The diagram highlights the RAF-MEK-ERK, PI3K/AKT, and JNK pathways that modulate cell protection and repair under control (*left*) and noise exposure (*right*) conditions. *GF* growth factor. **b**, **c** Levels of p-ERK1/2, p-AKT, p-JNK, and cleaved fragment f-PARP-1 were analyzed by Western blotting of cochlear lysates. A representative blot is shown in (**b**). Protein levels were normalized using total AKT levels, except for f-PARP1, which was normalized using the β-actin levels. Samples from at least three mice for each condition and genotype were tested. The average densitometric values are expressed as mean ± SEM. **c** The significance of the difference was determined using Student’s *t* test: ^+^ *P* < 0.05 versus non-treated wild type; ^++^ *P* < 0.01 versus non-treated wild type; ^+++^ *P* < 0.001 versus non-treated wild type; *P < 0.05 versus non-treated heterozygous; **P < 0.01 versus non-treated heterozygous. Exposure to noise increased cleaved f-PARP-1, ERK1/2, and JNK phosphorylation ratios, but not those of AKT

The phosphorylation state of key kinases was studied in the cochleae of wild-type and heterozygous mice 2 days after noise exposure (Fig. 8a). Heterozygous cochleae exhibited significantly higher baseline ratios of p-JNK/JNK than wild-type mice. In response to noise, both genotypes showed an increase of around 2-fold and 1.5-fold, respectively, in the activity ratios of p-ERK/ERK and p-JNK/JNK. The p-AKT/AKT ratio, an index of cell survival, was not significantly different between genotypes, but cleaved PARP (f-PARP-1) levels, an index of apoptotic cell death, were increased in response to noise (Fig. 8b, quantification in c). PARP fragmentation was significantly stronger in heterozygous than in wild-type cochleae.

## Discussion

RAF kinases play an essential role during embryonic development, as demonstrated by the fact that null mutants are not viable (*B*-*Raf* ^−*/*−^), have a high rate of embryonic lethality (*C*-*Raf* ^−*/*−^) or die shortly after birth (*A*-*Raf* ^−*/*−^) [13, 39, 58]. In this study, we show that the three RAF kinases are expressed in the mouse inner ear with similar developmental expression patterns. Each RAF kinase has highly specific functions [12, 21, 59] although they are exchangeable in certain contexts [20]. Our previous work showed that RAF kinases are targets of IGF-1, promoting cell proliferation, cell survival, and neuritogenesis during early development of the chicken inner ear. C-RAF inhibition in the chicken otic vesicle triggered apoptosis, whereas B-RAF and C-RAF inhibitions also impaired proliferation of the otic neuroepithelial progenitors [24]. Similar results have been reported in other species [60].

*C*-*Raf* null mice have poor embryonic survival rates, exhibit significant malformations at birth, including a reduced body size, and die at an early postnatal age because of extensive liver apoptosis [19]. In contrast, the middle and inner ears of null mice did not show any evident malformations or size reductions. Gross morphology and cytoarchitecture of the cochlea as well as expression of cell-type markers in the spiral ganglion and organs of Corti were similar in null and wild-type mice. We did not observe any compensation at the gene expression level of the other RAF kinases, and, accordingly, proliferation (ERK1/2) and survival pathways (AKT) showed normal activation levels in null mice cochlea. In contrast, we observed increased FoxG1expression, a transcription factor typically considered a target of the PI3 K/AKT pathway [61]. FoxG1 plays an important role in inner ear morphogenesis [54], and its expression is up-regulated in auditory neurons upon apoptotic insult [57]. RAF proteins have been shown to cross-talk with the PI3K/AKT pathway [61]. Therefore, it is entirely possible that the cochlea compensated for C-RAF developmental deficiency by basal activity of other RAF kinases and by cross-talk with basal AKT to up-regulate FoxG1 dependent cell survival. Further understanding of the cross-talk between FoxG1 and C-RAF would shed light on the role of these molecules in the development of cochlear structure and function.

*C*-*Raf* null mice exhibited a normal cochlear cytoarchitecture, but they had a profound bilateral sensorineural deafness that affected all frequencies. Deafness can be explained by reduced expression of the inwardly rectifying the K^+^ Kir4.1 channel in the stria vascularis. Kir4.1 is essential for K^+^ recycling, endolymph homeostasis, and maintenance of the endocochlear potential; hence Kir4.1 mutations cause human [62] and mouse deafness [63]. To our knowledge, there are no published data linking RAF kinases to Kir4.1 expression. Adapter 14–3–3 proteins bind C-RAF as well as several ion channels to modulate their function, but their potential binding to Kir4.1 has not been described [9, 64, 65].

Within the stria vascularis, Kir4.1 is expressed by intermediate cells which are melanocytes of neural-crest origin, whereas most of the other inner ear cell types develop from the otic placode [25]. This fact raised the possibility that these mice had a potential defect in melanocyte migration. C-RAF has been implicated in the migration of mouse keratinocytes and fibroblasts [66], migration of human endometrial stromal cells [67], and, together with B-RAF, in hair follicle melanoblast self-renewal [3]. However, melanocytes were present in the stria vascularis of null mice, although a defect in the renewal of melanocytes in null mice could account for their reduced recovery level from noise injury. Finally, the stria vascularis is highly rich in capillaries, and defects in the striatal microvasculature might affect the integrity of the surrounding cells and their molecules as it affects their functions [68, 69]. Endothelial defects are traditionally associated with *B*-*Raf* deficiency [58] but C-RAF is important for endothelial-cell survival during angiogenesis [70]. However, we discarded a generalized endothelial defect because KCNQ1 and Na^+^/K^+^-ATPase were expressed at normal levels in null mice.

C-RAF has a well-known role in cell survival, protection, and repair [5]; therefore, we decided to study the consequences of its chronic deficiency on the auditory receptor’s response to injury. *C*-*Raf* heterozygous mice showed a normal hearing threshold, and their cochlear morphology was similar to that of wild-type mice, but the JNK stress kinase basal activation level was increased, and the PARP-1-dependent DNA-repair mechanisms were impaired. Accordingly, 30 min of exposure to moderate noise caused widespread cellular damage in heterozygous mice cochleae, which was associated with an irreversible shift in the ABR threshold. Cellular loss was generalized and included outer hair cells, neurons, spiral-limbus fibrocytes, and type IV fibrocytes of the spiral ligament. The damage response pattern was entirely different in wild-type mice, which showed less cellular loss and a lower threshold shift that partially recovered over time. These results suggested that heterozygous mice had an increased susceptibility to noise-induced injury.

Noise exposure causes the activation of various biochemical pathways, including the JNK pathway [28], whose activation leads to apoptosis [71–73]. Accordingly, blocking apoptotic signaling using JNK inhibitors diminished the extent of cochlear damage and hearing loss caused by noise [74, 75]. Survival pathways are activated in noise-resistant mouse strains [76], and ERK phosphorylation is induced by mechanical damage and noise exposure [28, 77]. B-RAF is the main RAF kinase that promotes ERK phosphorylation and cell proliferation [78], and it is expressed in hair cells. Both heterozygous and wild-type mice showed similar increased p-ERK levels after noise exposure. These data suggest that B-RAF acts as the upstream kinase in the process of noise damage recovery, whereas C-RAF survival actions are mediated by an ERK-independent mechanism for which B-RAF cannot compensate.

Taken together, the results of this study strongly support the involvement of RAF kinases in repair and protection of the auditory receptors.

### Electronic supplementary material

Below is the link to the electronic supplementary material.

Supplementary material 1 (DOCX 15 kb)

Supplementary material 2 (DOCX 15 kb)
