# Supplementary material for: C-Raf deficiency leads to hearing loss and increased noise susceptibility
Source: Cell Mol Life Sci. 2015 May 15;72(20):3983–98. doi: 10.1007/s00018-015-1919-x (PMC4575698; doi:10.1007/s00018-015-1919-x)
Supplement: Supplementary file 1 — Supplementary material 1 (DOCX 15 kb) [file 18_2015_1919_MOESM1_ESM.docx]

***C-Raf* deficiency leads to hearing loss and increased noise susceptibility**

Rocío de Iriarte Rodríguez ^1,2*^, Marta Magariños^1,2,3* #^, Verena Pfeiffer^4,a^ , Ulf R. Rapp^4,b^

and Isabel Varela-Nieto^1, 2^

^1^ Instituto de Investigaciones Biomédicas “Alberto Sols”, CSIC-UAM, Arturo Duperier 4,

28029 Madrid, Spain.

^2^ CIBERER, Unit 761, Instituto de Salud Carlos III. 28029 Madrid, Spain

^3^ Departamento de Biología, Universidad Autónoma de Madrid, Darwin 2, 28049 Madrid, Spain.

#Corresponding author: Marta Magariños, [mmagarinos@iib.uam.es](mailto:mmagarinos@iib.uam.es)

**Online resource 1. List of antibodies and techniques used**

| ***Antibody*** | ***Type^1^*** | ***Source/Cat #*** | ***Concentration*** |
| --- | --- | --- | --- |
| **Anti-14-3-3ζ/δ D7H5** | RbP | Cell Signaling/7413 | 1:1000 (WB) |
| **Anti-β-actin** | MouM | Sigma/A5441 | 1:5000 (WB) |
| **Anti-Akt1/2** | GP | Santa Cruz/sc-1619 | 1:1000 (WB) |
| **Anti-phospho-Akt^Ser473^** | RbP | Cell Signaling/9271 | 1:1000 (WB) |
| **Anti-A-RAF** | RbP | Santa Cruz/sc-408 | 1:1000 (WB) |
| **Anti-B-RAF H145** | RbP | Santa Cruz/sc-9002 | 1:1000 (WB) |
| **Anti-phospho B-RAF** | RbP | Cell Signaling/ 2696 | 1:1000 (WB) |
| **Anti-C-RAF** | MouM | BD/ 610152 | 1:500 (WB) |
| **Anti-phospho C-RAF** | RbP | Cell Signaling/9427 | 1:1000 (WB) |
| **Anti-ERK MAPK** | RbP | Cell Signaling/9102 | 1:1000 (WB) |
| **Anti-phospho-ERK MAPK** | RbP | Cell Signaling/ 9101 | 1:1000 (WB) |
| **Anti-SAPK/JNK** | RbP | Cell Signaling/ 9252 | 1:1000 (WB) |
| **Anti-phospho-SAPK/JNK (Thr183/Tyr185)** | RbP | Cell Signaling/ 4668 | 1:1000 (WB) |
| **Anti-KCNQ1** | RbP | Santa Cruz/sc-20816 | 1:200 (IHC) |
| **Anti-Ki 67** | RbM | Master Diagnostica/0264-02 | 1:200 (IHC) |
| **Anti-Kir4.1** | RbP | Chemicon/AB5818 | 1:200 (IHC) |
| **Anti-myelin protein 0** | ChP | NOVUS Biologicals/NB100-1607 | 1:100 (IHC) |
| **Anti-myosin VIIa** | RbP | Proteus/ PT-25-6790 | 1:150 (IHC) |
| **Anti-Na^+^/K^+^- ATPase** | RbP | Upstate/06-171 | 1:400 (IHC) |
| **Anti-neurofilament** | MouM | Millipore | 1:100 (IHC) |
| **Anti-P38 MAP kinase** | RbP | Cell Signaling/9212 | 1:2000 (WB) |
| **Anti-phospho P38 MAP Kinase** | RbP | Cell Signaling/9211 | 1:5000 (WB) |
| **Anti-PARP-1** | RbP | Santa Cruz/sc-7150 | 1:1000 (WB) |
| **Anti-Sox2** | GP | Santa Cruz/sc-17320 | 1:50 (IHC) |
| **Anti-synaptophysin** | RbP | DAKO/IR776 | 1:100 (IHC) |

Summary of antibodies used for immunohistochemistry and Western blotting. Antibody type: RbP, rabbit polyclonal; RbM, rabbit monoclonal; MouM, mouse monoclonal; GP, goat polyclonal; ChP, chicken polyclonal. Abbreviations: IHC, immunohistochemistry; WB, Western blotting.
