# Supplementary material for: C-Raf deficiency leads to hearing loss and increased noise susceptibility
Source: Cell Mol Life Sci. 2015 May 15;72(20):3983–98. doi: 10.1007/s00018-015-1919-x (PMC4575698; doi:10.1007/s00018-015-1919-x)
Supplement: Supplementary file 2 — Supplementary material 2 (DOCX 15 kb) [file 18_2015_1919_MOESM2_ESM.docx]

***C-Raf* deficiency leads to hearing loss and increased noise susceptibility**

Rocío de Iriarte Rodríguez ^1,2*^, Marta Magariños^1,2,3* #^, Verena Pfeiffer^4,a^ , Ulf R. Rapp^4,b^

and Isabel Varela-Nieto^1, 2^

^1^ Instituto de Investigaciones Biomédicas “Alberto Sols”, CSIC-UAM, Arturo Duperier 4,

28029 Madrid, Spain.

^2^ CIBERER, Unit 761, Instituto de Salud Carlos III. 28029 Madrid, Spain

^3^ Departamento de Biología, Universidad Autónoma de Madrid, Darwin 2, 28049 Madrid, Spain.

#Corresponding author: Marta Magariños, [mmagarinos@iib.uam.es](mailto:mmagarinos@iib.uam.es)

**Table S2. Cochlear gene expression levels.**

| **GENE NAME** | **Fold change**  **qRT-PCR** |
| --- | --- |
| *FoxM1 (Forkhead box M1)* | 0.87±0.05 |
| *FoxG1 (Forkhead box G1)* | 2.54±0.58** |
| *Gap43 (Growth-associated protein 43)* | 0.89±0.17 |
| *Gmf-b (Glial maturation factor, beta)* | 0.94±0.07 |
| *Igf1R ( IGF-I high affinity receptor)* | 1.06±0.20 |
| *Mapk14(* *p38α,* *Mitogen-activated protein kinase 14)* | 1.07±0.13 |
| *Mash1 (Mammalian achaete-scute homolog 1) [Ascl1, Hash1]* | 1.22±0.61 |
| *Mef2D (Myocyte enhancer factor 2D)* | 1.13±0.08 |
| *Ntn1(Netrin1)* | 1.18±0.04 |
| *p27 kip1/Cdkn1b (Cyclin-dependent kinase inhibitor 1B)* | 1.28±0.16 |
| *Raf-A (Raf kinase A)* | 0.78±0.19 |
| *Raf-B (Raf kinase B)* | 1.11±0.11 |
| *Sox2 (SRY-box containing gene 2)* | 1.20±0.19 |

Expression levels of 13 cochlear genes in E18.5 wild type and null mice were compared using RT-qPCR. Gene-expression values were calculated as 2^–ΔΔCt^. Results were expressed as the fold-change ±SEM of the level in null versus wild type mice. Samples of at least three mice from each genotype were evaluated in triplicate. **P<0.005 versus wild type.
